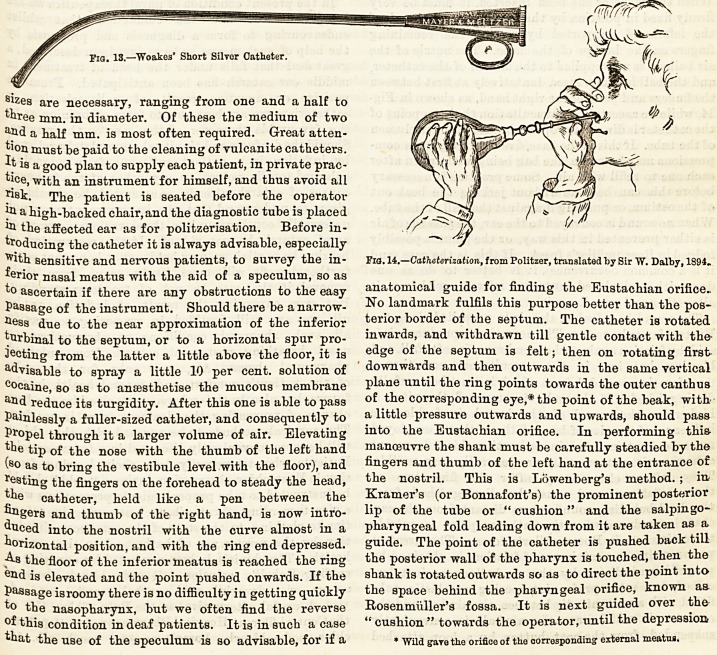# Chronic Catarrh of the Middle Ear.—V

**Published:** 1894-11-17

**Authors:** 


					Noy. 17, 1894. THE HOSPITAL. 115
Medical Progress and Hospital Clinics.
LThe Editor will be glad to receive offers of co-operation and contributions from members of the profession. All letters
should be addressed to Tele Editor, The Lodge, Porchester Square, London, ~W7]
CHRONIC CATARRH OF THE MIDDLE
EAR.?Y.
{Continued from page 483, vol. xvi.)
Catheter is ation has, in the writer's opinion, been
rendered a good deal easier to perform, and also more
endurable for the patient, sincev the introduction of
shorter catheters. With these instruments leverage
18 niuch reduced, and therefore le3s irritation is caused
the sensitive parts with which the beak comes in
contact. A length now in common use for silver cathe-
ters is twelve centimetres, or about four and three-
quarter inches. The general consensus of opinion is,
however, at present in favour of vulcanite as a better
niaterial than silver, since, on account of its flexibility,
*t occasions a minimum of discomfort to the patient,
^ho always prefers it. A useful length is about six
inches, with a curve at the beak of 145?, but this angle
can be altered by warming to suit each case. Three
Slzes are necessary, ranging from one and a half to
three mm. in diameter. Of these the medium of two
ai*d a half mm. is most often required. Great atten-
tion must be paid to the cleaning of vulcanite catheters,
^?t is a good plan to supply each patient, in private prac-
tice, with an instrument for himself, and thus avoid all
risk. The patient is seated before the operator
ja a high-backed chair, and the diagnostic tube is placed
111 the affected ear as for politzerisation. Before in-
troducing the catheter it is always advisable, especially
^ith sensitive and nervous patients, to survey the in-
ferior nasal meatus with the aid of a speculum, so as
to ascertain if there are any obstructions to the easy
Passage of the instrument. Should there be a narrow-
ness due to the near approximation of the inferior
turbinal to the septum, or to a horizontal spur pro-
jecting from the latter a little above the floor, it is
advisable to spray a little 10 per cent, solution of
cocaine, so as to ansesthetise the mucous membrane
arid reduce its turgidity. After this one is able to pass
painlessly a fuller-sized catheter, and consequently to
Propel through it a larger volume of air. Elevating
the tip of the nose with the thumb of the left hand
(?o as to bring the vestibule level with the floor), and
resting the fingers on the forehead to steady the head,
the catheter, held like a pen between the
hugers and thumb of the right hand, is now intro-
duced into the nostril with the curve almost in a
horizontal position, and with the ring end depressed.
the floor of the inferior meatus is reached the ring
end is elevated and the point pushed onwards. If the
Passage is roomy there is no difficulty in getting quickly
to the nasopharynx, but we often find the reverse
?f this condition in deaf patients. It is in such a case
that the use of the speculum is so advisable, for if a
septal crest (spur) be present, the catlieter?if passed
without its guidance, especially in the hands of a
novice?is almost sure to ride along its upper surface,
and when it arrives at the post nasal space, rotation
is found to be impossible.
Under these circumstances there is nothing to be-
done but to withdraw the catheter and reintroduce it,
taking care this time that the point of the beak is kept
in contact with the floor the whole way; In a difficult,
case it may be necessary, after cocainisation, to select
the smallest sized catheter, and slightly diminish the
curve of the beak before this can be done. The
writer does not remember having found occasion
to catheterise from the opposite side, but this
is said to be easy enough by those who employ
that method in this class of case. Supposing the beak
of the catheter to have been successfully passed into
the nasopharynx, it is necessary for those who are not
yet familiar with the procedure to have a definite
anatomical guide for finding the Eustachian orifice..
No landmark fulfils this purpose better than the pos-
terior border of the septum. The catheter is rotated
inwards, and withdrawn till gentle contact with the
edge of the septum is felt; then on rotating first
downwards and then outwards in the same vertical
plane until the ring points towards the outer can thus
o? the corresponding eye,* the point of the beak, with
a little pressure outwards and upwards, should pass
into the Eustachian orifice. In performing this-
manoeuvre the shank must be carefully steadied by the
fingers and thumb of the left hand at the entrance of
the nostril. This is Lowenberg's method. ; in
Kramer's (or Bonnafont's) the prominent posterior
lip of the tube or " cushion" and the salpingo-
pharyngeal fold leading down from it are taken as a
guide. The point of the catheter is pushed back till
the posterior wall of the pharynx is touched, then the
shank is rotated outwards so as to direct the point into
the space behind the pharyngeal orifice, known aa
Rosenmuller's fossa. It is next guided over the
" cushion " towards the operator, until the depression
* Wild gave the orifice of the corresponding external meatus.
Slzes are necessary, ranging from one and a half to
^ree mm. in diameter. Of these the medium of two
and a half mm. is most often required. Great atten-
tion must be paid to the cleaning of vulcanite catheters.
is a good plan to supply each patient, in private prac-
tice, with an instrument for himself, and thus avoid all
*isk. The patient is seated before the operator
jn a high-backed chair, and the diagnostic tube is placed
the affected ear as for politzerisation. Before in-
troducing the catheter it is always advisable, especially
^ith sensitive and nervous patients, to survey the in- Fia. 14.?Catheterization, from Politzer, translated by Sir W. Da!by, 1894.
ferior nasal meatus with the aid of a speculum, so as
to ascertain if there are any obstructions to the easy anatomical guide for finding the Eustachian orifice.
Passage of the instrument. Should there be a narrow- landmark fulfils this purpose better than the pos-
sess due to the near approximation of the inferior terior border of the septum. The catheter is rotated
turbinal to the septum, or to a horizontal spur pro- inwards, and withdrawn till gentle contact with the
jecting from the latter a little above the floor, it is edSe of the septum is felt; then on rotating first
advisable to spray a little 10 per cent, solution of downwards and then outwards in the same vertical
cocaine, so as to anaesthetise the mucous membrane Plane until the rin? points towards the outer canthus
and reduce its turgidity. After this one is able to pass oE tlie corresponding eye* the point of the beak, with
Painlessly a fuller-sized catheter, and consequently to a little pressure outwards and upwards, should pass
Propel through it a larger volume of air. Elevating into the Eustachian orifice. In performing this.
the tip of the nose with the thumb of the left hand manoeuvre the shank must be carefully steadied by the
(?o as to bring the vestibule level with the floor), and Angers and thumb of the left hand at the entrance of
resting the fingers on the forehead to steady the head, the nostril. This is Lowenberg's method. ; m
the catheter, held like a pen between the Kramer's (or Bonnafont's) the prominent posterior
Angers and thumb of the right hand, is now intro- lip of the tube or " cushion" and the salpingo-
dnced into the nostril with the curve almost in a pharyngeal fold leading down from it are taken as a
horizontal position, and with the ring end depressed. guide. The point of the catheter is pushed back ti
the floor of the inferior meatus is reached the ring the posterior wall of the pharynx is touched, then the
end i3 elevated and the point pushed onwards. If the shank is rotated outwards so as to direct the point into
passage isroomy there is no difficulty in getting quickly the space behind the pharyngeal orifice, known aa
to the nasopharynx, but we often find the reverse Rosenmuller's fossa. It is next guided over t e
of this condition in deaf patients. It is in such a case " cushion " towards the operator, until the depression
that the use of the speculum is SO advisable, for if a * Wild gave the orifice of the corresponding external meatus.
116 THE HOSPITAL. Nov. 17, 1894.
of the opening being felt it is directed into the latter
as before. This method entails the previous acquire-
ment of considerable tactile experience, and answers
best when the posterior lip is well developed.
In a perfectly straightforward case the routine
followed by those who are constantly employing the
catheter is exceedingly simple. The instrument is
guided along the floor until the upper inclined
plane of the soft palate is distinctly felt. Unless this
is reflexly irritated and contracted a good deal (as it
generally is by people who screw up their faces and
noses, the relationship between the position of the
palate and tube being thus completely altered),
the catheter, after gently clasping the velum with its
beak, needs only to be passed slightly further back
and then rotated outwards and upwards to the usual
elevation, when it will easily enter the tube. It is in
a difficult case, where some obstruction is present and
the results of auscultation are unsatisfactory, that
definite guides and methods applied with both
patience and skill are required. A great help
towards adeptness in catheterism is to pre-
viously learn posterior rhinoscopy so as to be quite
familiar with the topography of the nasopharynx.
When the catheter has been inserted, it must be very
firmly fixed in position by the forefinger and thumb of
the left hand, supported by resting the remaining
fingers on the bridge of the nose. The nozzle of the
air balloon is now applied to the mouth of the catheter,
and the ball is compressed tentatively at first between
the fingers and palm of the right hand, as shown in Fig.
14, while we ascertain by auscultation that the point of
the catheter is directed properly in the axis of the lumen
?of the tube. If this is the case, five or six vigorous com-
pressions may be made, the bag being withdrawn after
each one to refill with air. Some practice is necessary
before this can be done without jerking the beak out
of the ostium, or pushing it against the wall of the tube.
When no sound is conveyed to the ear, the passage of air
is either prevented in this way, or the point is possibly
lodged in Rosenmiiller's fossa. If this is the case (and
it is a common occurrence), it is better to do as one
does with a misplaced laryngeal mirrow?withdraw it
altogether and pass it again.
The sound heard when the air is propelled along the
Eustachian tube varies. The normal one, produced, it is
said, by friction against the membrane, is soft, full, and
blowing, but less in the auscultator's ear than a
" perforation sound," from which it must be carefully
distinguished. If the tube is stenosed the pitch is
higher, or the sound of the inrush is feeble; the
influence of the size of bore of the catheter employed
must be kept in mind in estimating this. In recent
catarrh moist mucous rales are generally very audible.
They often clear away after the first two or
three inflations, and must be distinguished from
the loud bubbling sound, more distant from the ear,
that is produced when the catheter point is outside
the ostium, or in Rosenmiiller's fossa. The greatest
power, owing to a minimum of friction, is obtained by
propelling air direct by the air balloon as just des-
cribed, but some prefer interposing an elastic tube
fitted with a vulcanite tip between the air-ball and the
catheter, in which case the air-ball is conveniently
suspended from the coat button by a loop attached
to its neck. There need then, of course, be no mis-
placement of the catheter, but it is better on the
whole to accustom oneself to the former method
from the first. We must, in either case, take care
that the vulcanite tip fits the catheter properly, and
the removal of the latter from the nose should
be effected deftly and painlessly; if it has been
correctly inserted, the grip of the ostium tubse will
be distinctly felt as this is done. After inflation, we
ascertain what effect this has had upon the hear-
ing distance for the watch and conversation. In
certain cases of chronic character it is common to find
audition in the first instance rather worse, owing to in-
creased tension, from imprisonment of an excess of air
in the tympanum after the Eustachian tube has
collapsed again. Swallowing a little water usually
allows the superfluous air to escape by degrees into
the pharynx. For removing the feeling of stuffedness
experienced after politzerisation, the patienb usually
appreciates the use of Siegle's speculum, or Del-
stanche's " rarefacteur." The temporary aggravation
of the deafness is by these means frequently followed
by improvement.
Treatment.
In the present condition of aural therapeutics we are
compelled to make the confession that whilst
endeavouring to form a diagnosis and prognosis by
the help of such means as have just been described, a
great deal that falls under the head of treatment in
middle ear catarrh has been anticipated. From the
positive or negative result of politzerisation or
catheterism, we are best able to form an opinion as to
the favourable character of the case or the reverse.
Should the deafness and tinnitus be completely relieved
by inflation in a patient suffering from adenoids,
enlarged tonsils, polypi, or any other of the frequent
causes of catarrh already enumerated, he should be
given to understand that the improvement may not
necessarily continue unless these various sources of
aural catarrh be got rid of. If the restoration of
hearing be partial, we may hold out a hope that some
further benefit will most likely ensue from their
removal if the case be not of too long standing.
But even supposing the result of inflation to be
nil, we are still justified in proposing to rectify
all such abnormal conditions as obstructions to
nasal breathing, post-nasal catarrh from enlarged
pharyngeal bursa, &c., not only for the sake
of the patient's comfort and general health in the
future, but also in the endeavour to give him the best
chance of preventing the deafness from increasing.
The surgical methods to be pursued with this object
will be more conveniently discussed in a future article.
Patients who temporarily lose their hearing from
simple Eustachian obstruction during a " cold " should
be instructed to take proper hygienic precautions with
a view to warding off catarrh, such as paying due atten-
tion to clothing, to the state of the cutaneous circula-
tion by the proper use of baths, and to the general
health. The rhinitis is best combated by spraying or
sniffing Dobell's solution, ft sodce bicarb., boracis'
aa gr. vi., glycerini acid carbol., mxii., aq. ad.
53. A useful alternative is the Pulv. Chlor. Co. of
the Central Throat Hospital Pharmacopoeia. R potass,
chlor., sodce bicarb., boracis aa ^ss., sacch. alb.
Nov. 17, 1894. THE HOSPITAL. 117
Dissolve a teaspoonful in 5 to 10 ounces of water at
95 deg. F. for each douche. Ungt. Eucalyptol should
afterwards he painted within the nostrils; or a solu-
tion of menthol in paroleine (20 per cent.) may he
insufflated into the nose and naso-pharynx with
Burroughs and Wellcome's " A " atomiser. A strong
solution of menthol is less irritating than a weak one,
because the ansesthetising effect is more rapid and
complete. A favourite remedy at the Central Throat
Hospital is the " Yapor Benzol " (Ibid), which, when
inhaled through the nose, produces a very soothing
effect: R benzol mxl., ol. cassise mii., mag. carb. levis.
gr. xx., aq. ad. 5j. One teaspoonful to be added to
a pint of water at 140 deg. F., and inhaled. Similar
in effect is the Yapor Benzoini Co., ft Tr. benzoini co.
5]"., aq. ferv. (140 deg. F.) ad. Oj.
(To be concluded.)

				

## Figures and Tables

**Fig. 13. Fig. 14. f1:**